# Temporally-differentiated biogenic carbon accounting of wood building product life cycles

**DOI:** 10.1007/s42452-020-03979-2

**Published:** 2021-01-10

**Authors:** Marieke Head, Michael Magnan, Werner A. Kurz, Annie Levasseur, Robert Beauregard, Manuele Margni

**Affiliations:** 1 CIRAIG, Department of Mathematical and Industrial Engineering, École Polytechnique de Montréal, P.O. Box 6079 Centre-ville, Montréal, QC H3C 3A7 Canada; 2 Pacific Forestry Centre, Canadian Forest Service, Natural Resources Canada, 506 Burnside Road West, Victoria, BC V8Z 1M5 Canada; 3 Département de Génie de La Construction, École de Technologie Supérieure, 1100 Notre-Dame West, Montreal, QC Canada; 4 Faculté de Foresterie, de Géomatique Et de Géographie, Université Laval, Québec, QC G1V 0A6 Canada

**Keywords:** Wood products, Biogenic carbon, Emission timing, Temporary carbon storage, Life cycle inventory, End-of-life

## Abstract

Although standards have identified temporary carbon storage as an important element to consider in wood product LCAs, there has been no consensus on a methodology for its accounting. This work aims to improve the accounting of carbon storage and fluxes in long-life wood products in LCA. Biogenic carbon from harvested roundwood logs were tracked using the Carbon Budget Model Framework for Harvested Wood Products (CBMF-HWP). Carbon flows through wood product manufacturing, building life and end-of-life phases, and carbon stocks and fluxes from harvest to the atmosphere were estimated. To cover the products commonly used in the Canadian building industry, a range of softwood products types, provinces and territories and building lifetimes were considered. In addition, policy scenarios were considered in order to model the effects of dynamic parameters through time as a policy target is reached. Most wood products have similar emissions profiles, though cross-laminated timber has higher sawmill emissions and oriented-strand board has higher initial post-demolition emissions. The region of construction is also predictive of the initial post-demolition emissions. Higher recycling rates shift materials from landfills into subsequent product systems, thus avoiding landfill emissions. Landfill decay rates are affected by climate and results in a large range of landfill emissions. The degree of postponement of end-of-life emissions is highly dependent upon the wood product type, region and building lifespan parameters. This work develops biogenic carbon profiles that allows for modelling dynamic cradle-to-grave LCAs of Canadian wood products.

**Supplementary information:**

The online version of this article (doi:10.1007/s42452-020-03979-2) contains supplementary material, which is available to authorized users.

## Introduction

The 2003 Good Practice Guidance of the Intergovernmental Panel on Climate Change (IPCC) introduced methodologies for the estimation and reporting of carbon stocks and fluxes in harvested wood products (HWP) [1]. Until then, it was assumed that the sum of carbon additions to the HWP pools from current harvest was equal to the sum of carbon losses from the wood products that were harvested in prior years and that the size of the total HWP carbon pool was constant [2]. Instead of tracking the details of the fate of harvested carbon, the IPCC made the simplifying assumption that inputs are equal to outputs, thus effectively treating the carbon from wood harvest as being instantly oxidised, ignoring any time delays and storage benefits associated with HWP. Carbon storage in harvested wood products in Canada delays the emission of greenhouse gases [3], and results in lower carbon biogenic carbon emissions from Canada’s forest products industry in national greenhouse gas reporting [4].

In life cycle assessment (LCA) of individual products, it is necessary to determine the impact of harvest on overall emissions. One simplifying assumption is net biogenic carbon neutrality, which assumes that carbon harvested is offset by a similar amount of carbon that is regrown in the forest resulting in a net zero impact on the greenhouse gas balance in the forest [5, 6]. However, there are several ways in which the carbon contained in harvested biomass is not necessarily cancelled out by an equal sequestration of carbon dioxide in biomass regrowth. The carbon neutrality assumption does not consider the time needed to regrow the forest and offset carbon emissions, as it may take years to decades to counteract the carbon that has accumulated in the atmosphere since the release of a greenhouse gas [7–11]. This time delay is very scale dependent: in the extreme case of a single stand, regrowth may require decades to centuries, while at the landscape level, annual regrowth may balance all harvest losses. The biogenic carbon balance should be better accounted for in LCA, by considering the carbon uptake and emissions throughout every life cycle stage from the forest to the end-of-life of a product.

Several authors have highlighted the need to incorporate the biogenic carbon of wood products in product LCA [7, 8, 12–14]. Most LCA guidelines and standards covering wood products now also tend to stipulate specific measures for biogenic carbon accounting. Of eight surveyed LCA guidelines and standards on greenhouse gas emissions and wood products [15–22], all but ISO 14067 [15] take the position that biogenic carbon uptakes and emissions should be accounted for in LCA. However, they only provide the very simplified assumption that the uptake of carbon in forest should be considered a negative emission (i.e. removal) and that the release of carbon should be considered a positive emission. In the case of certain long-life products such as building materials, the carbon contained in the wood is stored for the duration of the building product life, which can amount to delaying emissions for several decades or centuries in some cases. In addition, the long-term storage of carbon in landfills has been identified as having potential climate benefits [23]. One of the main critiques of the neutrality assumption is that it ignores the questions of temporary carbon storage and delayed emissions, which can result in potential climate benefits. The storage of carbon in products is currently not considered in many LCA studies as there has been no consensus on how to account for it [24].

A few authors have developed methodologies to address the storage period of harvested wood products in the anthroposphere [25–27]. These authors all apply an aggregated method, known as GWP_bio_, that considers carbon dynamics throughout the life cycle of a biomass product and it relates those to the global carbon cycle. While these methods are useful for the specific contexts for which they are designed (i.e. knowing the rotation period of the harvested forest, oxidation of biomass upon the end of life), they do not allow for the adaptation of carbon dynamic profiles to the local context, such as Canadian managed forests and landfilling as a primary end-of-life waste treatment.

Forestry science has been considering the carbon balance of harvested wood products throughout their use phases for a few decades [3]. Brunet-Navarro et al. [28] reviewed 41 wood product models and classified them based on their functionality and performance. A wood product model can either estimate and evaluate the fate of biogenic carbon in different wood product classes or it can be used to estimate the carbon emissions from wood product use and end-of-life [28]. From a single wood product perspective, the latter is most relevant and requires a model that can track carbon, including the allocation of co-products, the consideration of time and the ability to handle various end-of-life treatment options. Such a carbon accounting model could be used in life cycle assessment to consider biogenic carbon storage and fluxes in wood products.

The objective of this study is to improve the biogenic carbon accounting of long-life wood products in LCA, by tracking biogenic carbon from harvested roundwood logs through wood product manufacturing, building life and end-of-life phases, by considering the carbon fluxes between the wood product and the atmosphere as temporally-differentiated life cycle inventories. This tracking is also used to test dynamic inventories through the use of policy scenarios that increase recycling rates and landfill gas collection. This improvement of biogenic carbon accounting in wood products will provide building designers with a more accurate portrait of the climate impacts of wood products, and support more informed decisions related to material selection. While the proposed method is applicable to any geographical region, in this paper the method is applied to the Canadian building context. To cover the products commonly used for structural elements in the Canadian building sector, seven types of softwood products, across 12 Canadian provinces and territories with building lifetimes varying from 0 to 150 years, are considered.

## Methods

### Wood product model

A team at the Canadian Forest Service [4] developed the Carbon Budget Model Framework for Harvested Wood Products (CBM-FHWP). CBM-FHWP allows for the dynamic construction, validation, simulation and analysis of a system that describes and quantifies the flow of carbon in harvested wood products through time and space. Within this flexible framework, users must define all aspects of the models they create, which includes the definition of the space (i.e. the origin of the harvested wood and the region of product use), the carbon stocks, the physical state, the mass of carbon, the flows as well as the model time step size. Until now the model has been mostly used for tracking the carbon of harvested wood products from a macro perspective for different geographical regions [29]. CBM-FHWP is currently most extensively used by the Canadian Forest Service to calculate the contribution of harvested wood products to Canada’s greenhouse gas balance for the national inventory reports submitted to the UNFCCC every year [4]. The specific perspective of this research project, with its focus on individual wood products throughout their life cycles, will be a new application of the modelling framework.

### Model scope and system boundaries

**Fig. 1:**
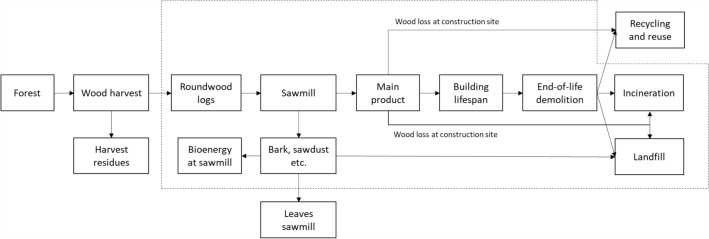
System boundaries for wood product carbon flows. The processes contained within the dotted line are included in the model. The forest ecosystem and upstream forest harvest activities are developed in a previous study [31]. Our implementation of the model treats the “Leaves sawmill” and “Recycling and reuse” processes as being outside of the system scope. The figure only includes the biogenic carbon contained within the wood

In all, seven wood product models (lumber, plywood, glulam, oriented strand board (OSB), laminated veneer lumber (LVL), cross laminated timber (CLT) and I-joists), which correspond to products or construction materials that would commonly be used in the construction of buildings in Canada, were built and simulated in CBM-FHWP. The models consider carbon from the roundwood log delivered to the sawmills, the sub-division of logs into products, the use of the co-products (use in bioenergy, external manufacturing or disposal), the storage of the wood carbon over the lifetime of the wood product, and the end-of-life processing, including a half-life approach for modelling the fate of the degradable carbon in landfills (Fig. 1). While some research [30] has indicated that storage wood chips, sawdust, etc. can result in non-negligible greenhouse gas emissions, these emissions are considered to be part of the co-product life cycles and thus are not included within the system boundaries. These models were run for building life years from 0–150 years^1^ for 12 Canadian provinces and territories (excluding Nunavut, for a total of 2352 cases). The model is focused on the biogenic carbon contained in the roundwood log input required for a given wood product, and as such, other wood product life cycle emissions are already included in life cycle inventory databases and thus they are not considered in this study.

The focus of this research is on creating temporally-differentiated biogenic carbon profiles for the life cycle of wood construction materials used in buildings. As such, the outputs of this work are in the form of life cycle inventories that can be subsequently used in life cycle impact assessment.

### Creating the model files and parameter definition

**Fig. 2:**
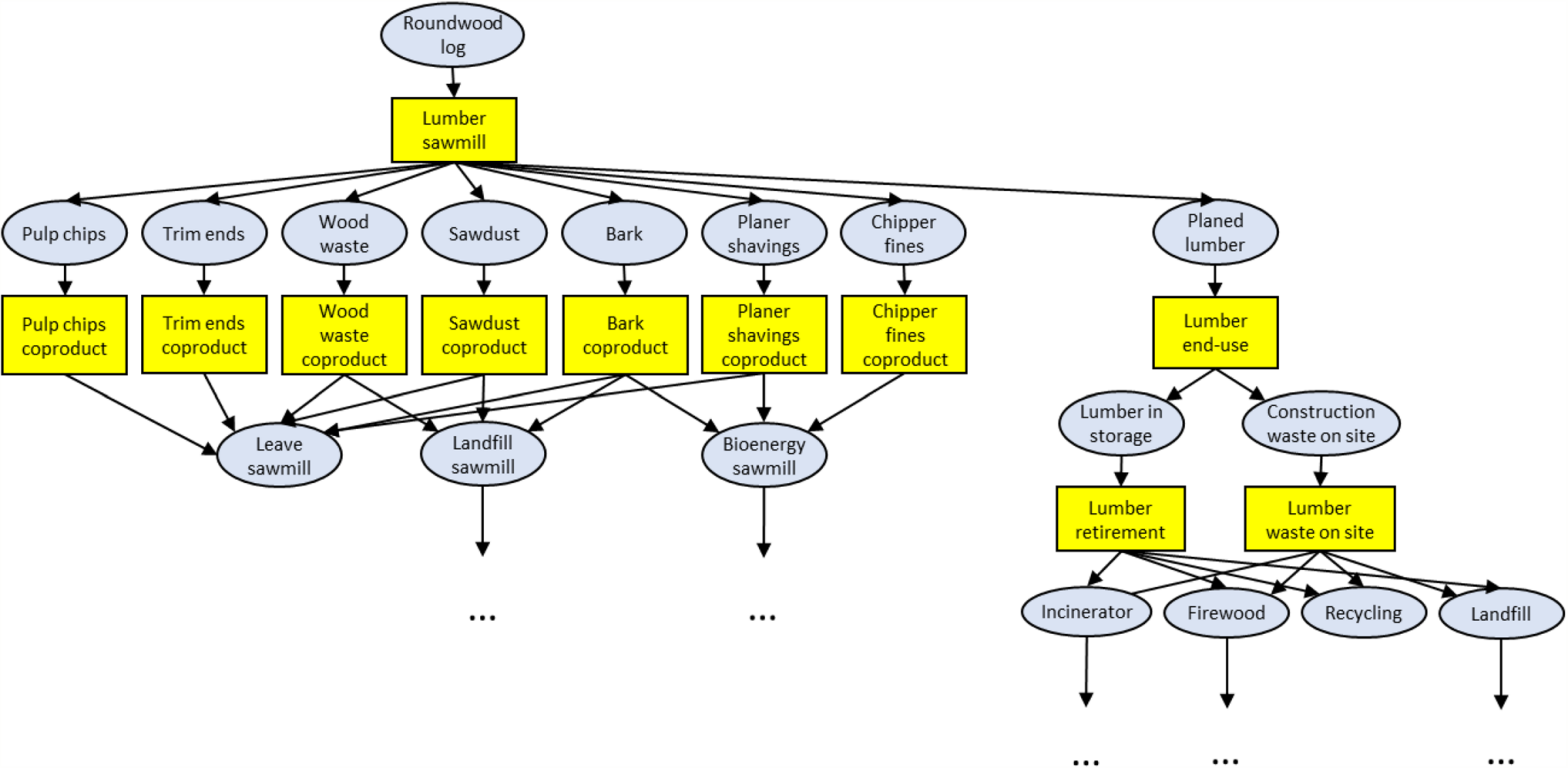
Excerpt of model structure for lumber products. Yellow squares indicate events and blue circles indicate pools. Dotted lines indicate where this model structure continues beyond shown excerpt

In CBM-FHWP, products are modelled as a series of text file line entries in six dimensions (space, stocks, physical states, mass, flows and time) [32]. At the flow level, *pools* and *events* are defined such that carbon can move through each life cycle and each co-product in succession. The partitioning of co-products at each event is done as proportions of a total of 100%. Figure 2 shows an excerpt of the model structure for lumber products.

Seven separate models were developed for each of the seven wood products, each model using the lumber model as a template. Each of the models begins with roundwood logs as input but has different co-product outputs and fates at the manufacturing (sawmill) phase. Mass balances of the seven wood products were obtained from Athena Sustainable Materials Institute reports [33–43]. The mass balances are provided as green wood and oven dried wood, and therefore a unit conversion step was required to account for carbon content. The carbon content for each pool was then calculated as a proportion of all pools flowing in or out of an event (Table 1).

**Table 1:** Co-product outputs of sawmills for seven wood product types (% mass flows)

	Lumber	CLT	Glulam	I-joist	LVL	OSB	Plywood
Main product	43.1%	54.0%	50.3%	55.0%	47.3%	79.3%	49.8%
Bark	8.9%	9.0%	8.7%	6.7%	11.3%		
Planer shavings	6.3%		2.9%	2.1%			
Sawdust	5.6%	4.4%	4.8%	1.9%			
Pulp chips	34.5%	32.5%	32.5%	21.1%	28.7%		19.4%
Trim ends	0.6%		0.3%	0.2%			
Chipper fines	0.2%	0.2%	0.2%	0.1%			
Wood waste	0.7%		0.3%	0.3%		0.3%	
Off-spec				2.4%	2.6%		
Peeler cores				3.4%	10.1%		9.0%
Wood for hog fuel				5.8%		17.4%	21.5%
By-products				1.0%		2.9%	
Veneer							0.3%
Total (log) %	100.0%	100.0%	100.0%	100.0%	100.0%	100.0%	100.0%
Total (log) kg C m^3^	421 kg C	390 kg C	415 kg C	360 kg C	375 kg C	236 kg C	288 kg C

*CLT* cross-laminated timber, *glulam* glue laminated timber, *I-joist* engineered wood joist, *LVL* laminated veneer lumber, *OSB* oriented strand board, *off*-*spec* off-specification, *by*-*products* unspecified co-products, *Total* (log) total roundwood log mass by % and kg C/m^3^ Source: [33–43]

Beyond the manufacturing phase, the structure of the models was identical for the use phase and end-of-life waste management of the main wood product. At the building construction site, the lumber is again divided into two co-products: the main building product and the waste occurring at the construction site. The amount of waste occurring at the construction site is taken from Wang et al. [44] as a waste factor of 10% at construction sites in North America. The remaining carbon (90%) is assumed to be embedded in the building. The wood remaining in the building is modelled as 100% in the building for every year up until the designed building life year, at which point 0% remains in the building and the carbon is moved to end-of-life treatment.

Both the construction site waste and the building demolition waste are treated via the same four end-of-life treatment options: landfilling, incineration, recycling and use as firewood. In Canada, the majority of construction wood waste is landfilled, with a small proportion being recycled despite specific municipal and provincial policies discouraging the landfilling of construction waste [45]. The proportions of waste going to different treatment options were based on an Environment Canada report on construction waste [46]. The proportion of wood recycled varies significantly between provinces, with 0% going to recycling in certain provinces and up to 50% in Nova Scotia. Detailed proportions going to recycling and landfill per province are given in Supplementary Material 1.

### Treatment of outputs from system

There are a few places in this model where outputs are utilised in other processes, such as the production of bioenergy and the use in other material life cycles (Fig. 1). When a process produces more than one useful product, it can be termed multifunctional. As such, only the flows directly related to that product in question must be accounted for in the calculation of its environmental impacts. ISO 14044 recommends a specific hierarchy for solving for multifunctional processes [47]. As a first priority, subdivision should be attempted, by dividing black box processes into single operation unit processes. Second, substitution should be attempted by either expanding the system boundaries to include another function that is not within the product system or by subtracting an alternative production process. Third, if neither subdivision or substitution is possible, the allocation of process burdens can be done by partitioning the process flows according to some chosen criterion. There is a preference for a physical means of allocation such as allocation by mass, energy content, stoichiometry, etc. However, in some cases allocation by economic value is appropriate.

The literature shows that the methodological choices surrounding multifunctional systems, particularly wood and forestry products, can have a significant impact on LCA scores [48–50]. For this research work we applied the guidance provided by the European EN16485 product category rule standard (Round and sawn timber—Environmental Product Declarations—Product category rules for wood and wood-based products for use in construction), which recommends allocating biogenic carbon according to the carbon content of the product.

A variety of different co-products were modelled as outputs at the manufacturing stage for the modelled wood products, including bark, shavings, sawdust, pulp chips, trim ends, chipper fines, peeler cores and off-specification product. The Athena Reports [33–43] specify end uses of all these co-products in a Canadian context, and we further streamlined these into three different fates:

Leaves sawmill: This refers to co-products that are sold off to other facilities to be used as a raw material. Given that these co-products (and their carbon content) are used by third parties, the carbon in the co-product is allocated to other systems (cut-off from the main product system) and also shares the burden of the processes with the main product.

Landfilling: The landfill fate is modelled as an average Canadian landfill as the Athena reports have not specified the geographic locations of all sawmills. The specifics of the treatment of landfills will be described with the other end-of-life options for the wood emerging from building demolition. The carbon released from landfilling sawmill co-products is allocated to the main wood product.

Bioenergy: The co-products can also be used for bioenergy at the sawmills. The bioenergy transforms the carbon embedded in the co-product into CO_2_ and (negligible) CH_4_ emitted from the combustion of the material. Carbon emitted through the combustion of co-products for bioenergy at the sawmill is allocated to the other wood co-products.

The proportions of each co-product that is going to each fate is provided in Supplementary Material 1.

### End-of-life waste management

#### Landfilling

Wood accounts for around 7% of all unrecovered waste sent to landfill in Canada [51]. Conditions within modern landfills are predominantly anaerobic due to their design both in preventing moisture and precipitation from entering the landfill and in the use of cover materials to prevent exposure to air. Typically, only a minimal amount of aerobic decomposition occurs, in cases when waste is not immediately covered [52]. Although the anaerobic decomposition of organic materials emits greenhouse gases [52], several studies [23, 44, 53–57] demonstrate that wood degrades very slowly in landfill sites. Since wood consists of a complex lignin matrix that integrates cellulose and hemicellulose and the conditions of most landfills are anaerobic, only a small proportion of wood is degraded.

According to Micales, Skog [56], it is estimated that only 0–3% of carbon contained in wood is emitted as a gas at landfill sites. Wang et al. [23] compared wood degradation of several types of wood products in laboratory-scale landfills for 440–1347 days until methane production could no longer be detected. For most wood types, degradation calculated as carbon conversion percentages, ranged between 0 and 7.9% (degradation for hardwood OSB was 19.9%). More recently, Wang et al. [44] found through field studies in the United States that the degradation of wood in a landfill is dependent upon the type of wood product. They found that after leaving wood in the landfill for 1.5–2.5 years, 5–23% of the carbon contained in the wood is degraded for engineered wood such as oriented-strand board (OSB), whereas for hardwood and softwood lumber very little (0–9%) of the carbon was degraded. In a study examining wood degradation in landfills in Australia, Ximenes et al. [55] found that temperate species experienced only 0–8% carbon loss after 16–44 years of being recovered.

Taking into consideration the variation of wood degradation in landfills found in the literature, which varies across wood types, wood species and local climatic conditions, we elected to make use of the landfill models supported by the CBM-FHWP. The CBM-FHWP models the degradation of carbon in landfills using the first order decay method, a method used by the IPCC [58]:

1 $$DDOCm = DDOCm_{0} \cdot e^{ - kt}$$

where *t* is time (years), *DDOCm* is the mass of the degradable organic carbon that will decompose under anaerobic conditions in a landfill at time t, *DDOCm*_*0*_ is the mass of DDOC at time 0, *k* is the decay rate constant (years^−1^).

Since the decay rate constant, k, is influenced by several factors such as climate, landfill engineering and waste composition, it is difficult to obtain values that are specific to both province/territory and wood type (lumber, OSB, etc.) [59]. Two sets of k values were used to model the degradation of carbon in landfills, 1) a value of 0.03 years^−1^ for average wood landfilled in Canada and 2) specific k values for each province and territory ranging from 0.003–0.083 years^−1^ [60]. A detailed table of these values is provided in Supplementary Material 1. The degradable organic carbon has three possible fates for the landfill gas resulting greenhouse gas emissions: capture of CH_4_ without flaring for energy generation (16.8%), capture of CH_4_ with flaring and direct emission of CO_2_ (17.2%) and direct release of landfill gas to the atmosphere (66%). The proportion of carbon emitted as CO_2_ and CH_4_ emissions from the combustion of landfill gas for energy production (capture without flaring) was modelled as 99.995% and 0.005%, respectively [61]. Carbon emissions from capture with flaring are 99.7% CO_2_ and 0.3% CH_4_ [60] and direct release of landfill gas yields 10% CO_2_ and 90% CH_4_ [58].

#### Recycling

The possibility for recycling construction wood waste in Canada is highly dependent upon the population density of the city or region. Larger urban centres are more likely to have a higher capacity for construction waste recycling than smaller cities or rural areas, where the economics of recycling these waste materials is unfavourable [46]. Recycling rates are also dependent upon the end-of-life classification of the wood. Solid (or untreated) wood tends to be recycled and have higher market values than engineered and treated woods that can contain adhesives, paints and preservatives [46]. However, the consequences for the carbon accounting of the reuse and recycling of wood can become complex, due to the way in which the emissions benefits of recycling are treated.

In terms of the climate implications of wood recycling, in particular, a few authors have published on this topic. In their study on the LCA of particle board, Wilson [62] considers carbon storage in the carbon balance and predicts that recycling processes will keep carbon out of the atmosphere even longer than the service life of the initial product. However, Werner et al. [63] conclude in their study on end-of-life alternatives of wood products that there is no method of modelling post-consumer wood that would account for all situations of wood use. Kim, Song [64] used system expansion to deal with recycled materials in particle board manufacturing, thus accounting for the avoidance of virgin materials through the use of recycled wood. They also calculated the carbon benefit of recycling based on the carbon storage of wood during the service life of the wood, as well as the extended period of storage attained through recycling. This calculation also accounts for the effects of progressively diminishing storage through material degradation as a result of several rounds of recycling. Although the approaches used for the treatment of wood recycling differ, it is clear that the recycling of wood can have significant implications in terms of biogenic carbon accounting.

The carbon content of the demolition wood sent to recycling is tracked, however in the model it is treated with a cut-off approach. The cut-off approach, whereby the subsequent fate of the recycled material is excluded from the scope of the system, was chosen for recycling in this study for a few reasons. First, the third parties purchasing the recycled wood material could be using it for a multitude of purposes and thus it could be substituting a variety of intermediary materials that would otherwise be made with virgin materials. Second, the timing of the ultimate disposal of the material as well as the number of product life cycles that the wood will be part of is unknown. Third, the carbon becomes part of another product, the “responsibility” for which belongs to that product life cycle. Finally, since the objective of this study was to provide temporally-differentiated life cycle inventories, the inclusion of the effects of subsequent life cycles would necessitate a full life cycle impact assessment (LCIA) and thus go beyond the scope of this work. However, choosing a cut-off approach may have a few implications in terms of the carbon fluxes attributed to the primary product life cycle and how these carbon emissions are characterised as climate impacts. By not accounting for this carbon at the point that it leaves the primary product life cycle, certain attributes of the future material use are not considered. For example, the wood could be sold to a waste management company to be chipped and used as a daily landfill cover. Aside from the financial transaction having taken place, there is very little difference to this fate than if the material had simply been landfilled. Nevertheless, this approach has been chosen for its flexibility, as it would allow solving for multifunctionality in post-processing calculations as a part of life cycle impact assessment.

#### Incineration and use as firewood

Incineration only accounts for a very small proportion of waste management in Canada [46], taking place in only a few municipalities. A quick survey of four specific municipalities, showed that the incinerators do not even accept construction and demolition waste. As such, 0% of wood waste is assumed to be incinerated in the model. However, in order to consider incineration either for additional regions outside of Canada or to investigate the impacts of potential incineration in Canada, incineration has been left as a waste management option in the model. The carbon emitted by the incinerator is modelled in this work to be mostly CO_2_ (99.999905%) with a negligible amount of CH_4_ (0.000095%) [65]. Since the heat created by incinerators can be harnessed for energy purposes, some incinerators generate usable electricity or heat. If desired, this could be accounted for separately.

Another waste management option included in the model is the potential for wood waste to be used as residential firewood. While construction wood waste is not treated via this method throughout municipalities, the option is left in the model for potential marginal cases, such as in remote areas, where individuals use construction wood waste as firewood. The carbon emitted by using waste wood as firewood, modelled as emissions from residential conventional stoves and fireplaces for CO_2_ (97% of C) and CH_4_ (3% of C) [66]. The combustion of firewood creates heat, which can substitute directly or indirectly for other residential heat sources, such as electrical, oil or natural gas heating systems. This substitution can be accounted for outside of the FHWP model if this type of waste treatment is used.

### Additional scenarios

In the context of buildings and construction, the eventual end-of-life phase of a building product can occur several decades in the future. In the meantime, proportions of waste going to different treatment options, and the waste management technology itself can evolve significantly, at which point the waste scenarios for demolished building materials is unknown. Sandin et al. [49] examined the effects of future waste management scenario assumptions on the outcomes of environmental impacts of building materials. Their results suggest that the assumptions made about waste management scenarios of the future, such as type of disposal, level of technology and type of LCA approach (attributional vs. consequential), may have significant impacts in terms of the relative environmental impacts of the end-of-life phase of material alternatives.

Some additional policy scenarios were considered which involve varying a key parameter through time as a policy target is reached, starting from the year 2020. These scenarios use existing base cases with static parameters and add the carbon emissions related to material or fuel substitution.

The scenarios are defined as:

*REC70%:* This scenario models the 70% construction waste recycling target by 2025 set by the Quebec government [45]. The recycling rate increases at the rate calculated based on construction waste recovery in Montreal, the city with the largest population in Quebec [67].

*LFG80%:* This scenario models an 80% landfill gas capture rate, based on the 75% capture rate by 2020 set by the British Columbia government [68]. Given the explicit targets for 75% LFG capture by 2020, we have assumed that an average of 80% capture rate by 2030 is possible, given that there is an average of 7 years of life left in British Columbia landfills [69].

The scenarios are run for only a select set of case parameters in order to show the full range of results without rerunning all 2352 cases (see Table 2).

**Table 2:** Base case parameters for testing end-of-life policy scenarios for wood products

Parameter	Low	Medium	High
Engineered wood product content (EWP)	Lumber (0% EWP)		I-Joist (100% EWP)
Recycling rates (provinces/territories)	Northwest Territories (0%)	Ontario (16–24%)^a^	Nova Scotia (40–51%)*
Landfill half-lives (provinces/territories)	British Columbia (8.3 y^−1^)	Saskatchewan (57.8 y^−1^)	Northwest Territories (346.6 y^−1^)
Building life	1	50	150

^a^depending on type of wood product and whether wood is from a construction or a demolition site

### Running the simulations

The default procedure for running results for a given set of parameters, involves using batch files that call up the CBM-FHWP software to convert the files to a usable format, import them and then run the simulation. In the case that several sets of parameters need to be run at once, CBM-FHWP is flexible enough to easily allow for customisable runs and thus can process several parameters sets at once. The scenarios were run and the results exported into a spreadsheet, to facilitate analyses. The results are calculated in terms of carbon transfers between HWP carbon pools, along a time scale from 0 to 300 years. The simulation period for the time scale presented was chosen for a few reasons. First, it allows for the modelling of the longest building lifespans (150 years) as well as sufficient time (an additional 150 years) for end-of-life emissions to propagate and eventually diminish to negligible quantities. Second, a consistent simulation period reduces the number of permutations of parameters that need to be simulated. The model output as carbon fluxes (kg C m^−3^ product) are provided in Supplementary Materials 3–9.

## Results

**Fig. 3:**
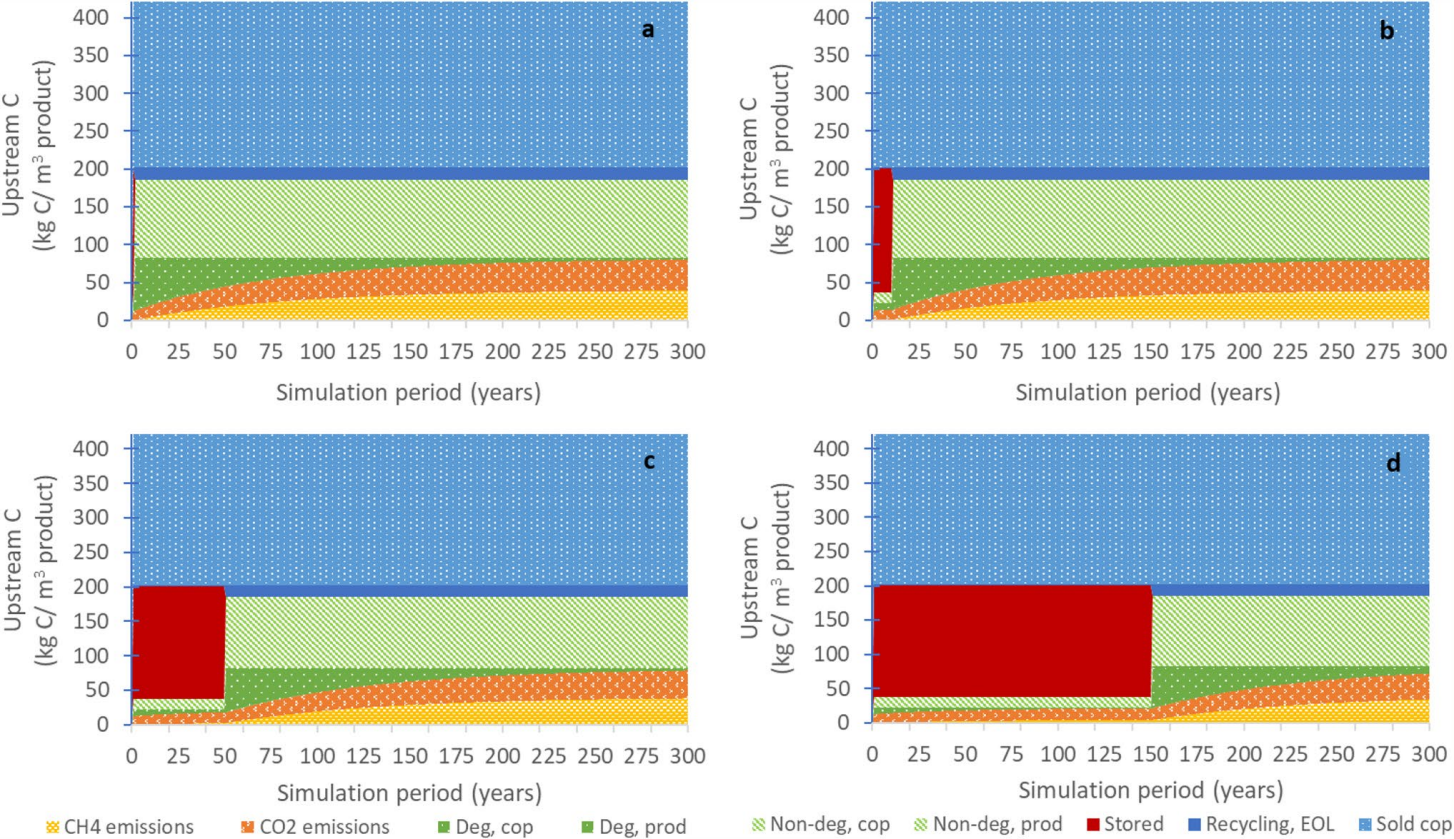
Change in carbon pools over time, for 1 m^3^ lumber, Alberta, for four buildings lifespans **a** = 1 year, **b** = 10 years, **c** = 50 years, **d** = 150 years). Deg, prod/cop = degradable portion of carbon in landfilled lumber main product/sawmill co-products, Non-deg, prod/cop = non-degradable portion of carbon in landfilled main product/sawmill co-products. Stored = carbon stored in building, Recycling, EOL = recycling of wood at end-of-life, Sold cop = co-products at sawmill sold to third parties

Tracking carbon in different carbon pools through time can be complex, especially in combination with variable building products, provinces and buildings lifespans. The fate of the carbon contained in the original roundwood logs input into the sawmill is illustrated through all carbon pools over 300 simulation years for four different building lifespans (Fig. 3).

Roughly 50% of the carbon of the roundwood log needed for the production of 1 m^3^ of lumber is transferred to other uses through the sale of sawmill co-products as for example, fibreboard, or through post-sawmill recycling processes (Fig. 3). The building life, which is represented by the amount of carbon stored in the product (in solid red), affects how much the end-of-life processes, including the storage of carbon in landfill and CO_2_ and CH_4_ emissions are delayed. Within the simulation period of 300 years, a longer building lifespan means that overall fewer cumulative C emissions from the wood product manufacture and end-of-life are released within the assumed time horizon. If for example, a typical 100-year simulation period had been chosen, the end-of-life emissions at demolition for a building lifespan of 100 + years would be entirely excluded as the simulation period would end prior to the release of these emissions. As such, the choice of the time horizon is key to determine the life cycle carbon emissions.

**Fig. 4:**
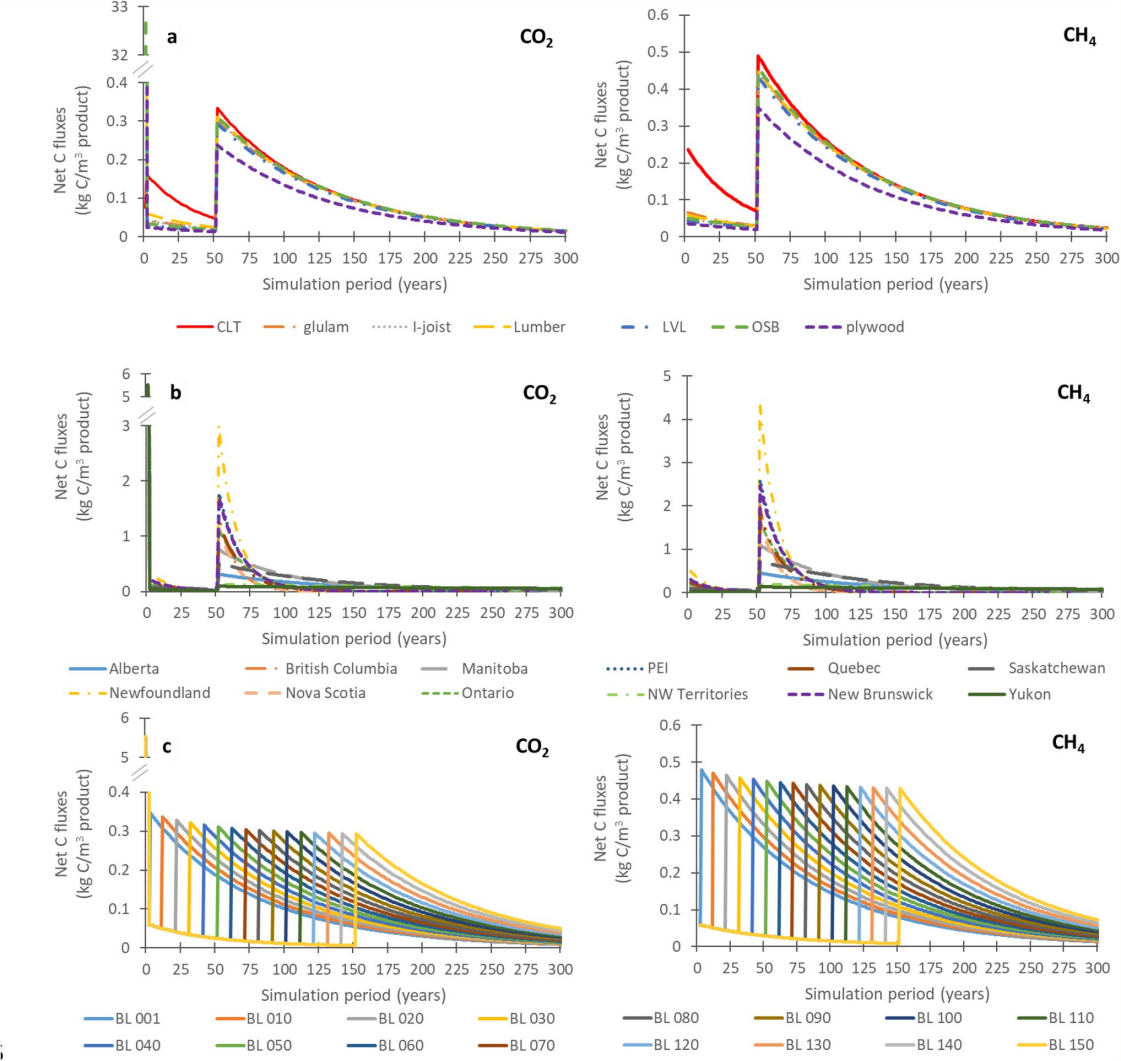
Net carbon fluxes (kg C m^−3^ product year^−1^) of CO_2_ and CH_4_ across: **a** seven building products (Alberta, building life = 50 years), **b** provinces (lumber, building life = 50 years), **c** building life (BL) years (Alberta, lumber)

The carbon emissions from manufacturing, use and end-of-life can vary as a function of wood product type, province or territory and building lifetime (Fig. 4).

**Fig. 5:**
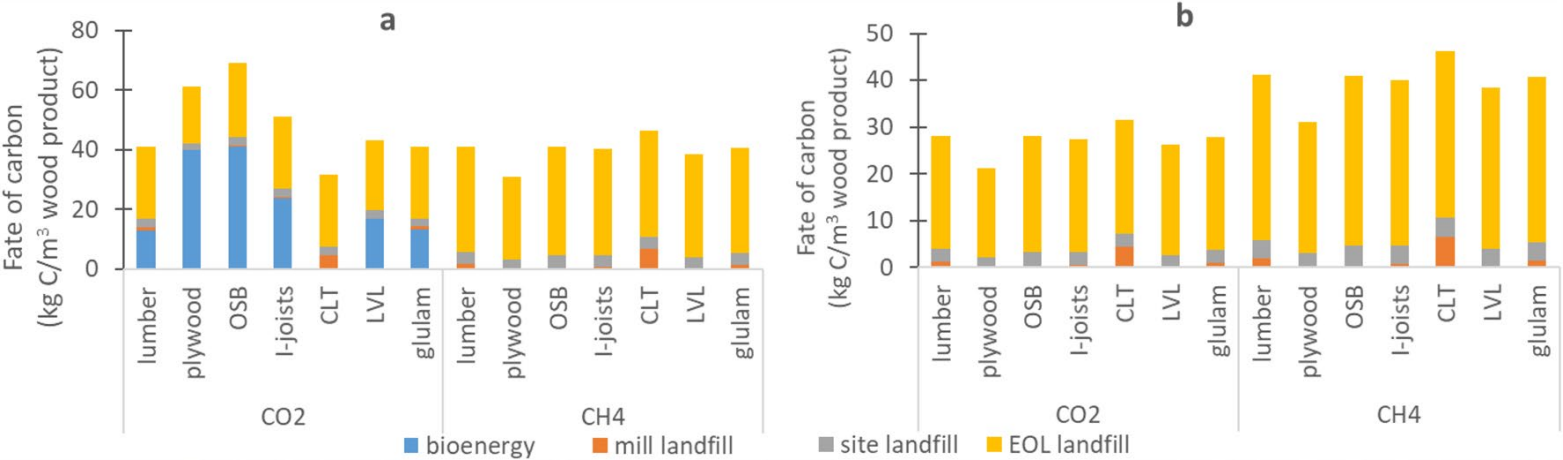
End fates of carbon for seven wood products at the sawmill (bioenergy and mill landfill), at the construction site (site landfill) and end-of-life (EOL landfill). **a** shows all end-of-life emissions, while **b** shows just landfilling emissions. The EOL landfill proportion are calculated using landfilling rates for Alberta (91.5%). The wood (as carbon) sold from the sawmill is cut-off from the system and is not considered here, and differs by wood type (in kg C m^−3^ (% of total roundwood logs): lumber = 219 (52%), plywood = 105(36%), OSB = 7 (3%), I-joists = 151 (42%), CLT = 178 (46%), LVL = 181 (48%), glulam = 214 (52%))

As shown in Fig. 4a, the highest emission pulses (reaching 32.7 kg C m^−3^ product for OSB) occur in year 1 due to sawmill co-product treatment via mostly biofuel and landfilling and construction site waste landfilling. Figure 5 shows the contribution of these emissions to the total life cycle carbon emissions, for both CO_2_ and CH_4_. When emissions results are examined across wood product types (Fig. 4a), CLT stands out in terms of the year 1 emissions, especially CH_4_ emissions, due to a larger proportion of the finished product (13%) that is deemed to be off-specification and thus is sent to landfill. In terms of the initial post-demolition emissions (year 50), most wood products cluster between 0.29 and 0.333 and 0.40–0.49 kg C m^−3^ product, for CO_2_ and CH_4_, respectively. The only wood product that does not follow this trend is plywood, with demolition year emissions of 0.24 and 0.35 kg C m^−3^ product, CO_2_ and CH_4_, respectively. The reason for this difference is related to lower proportion of roundwood log inputs at the sawmill going to the main product (43% for plywood vs. 44–79% for the other wood products) and not the co-products. Given the type of wood product, this makes sense as plywood is made of layered veneer, which makes it less forgiving in terms of product specifications and thus more co-products are produced at the manufacturing stage. A more detailed emissions contribution by life cycle stage is shown in Fig. 5, which shows the amount of carbon (in kg C m^−3^ wood product) going to various end-use fates for CO_2_ and CH_4_, for a) all emission outputs and b) all landfill outputs.

For a given wood product (lumber), the region in which the material is demolished and treated at end-of-life also affects both the CO_2_ and CH_4_ emissions occurring at and beyond the demolition year (Fig. 4b). This is a function of two factors: both the percentage of materials recycled as opposed to landfilled and the degradation rates of wood in the landfills (related to the climate of the region). For example, emissions are particularly high (3.0 and 4.4 kg C m^−3^ CO_2_ and CH_4_, respectively) in the case of lumber disposal in Newfoundland due to the combination of a higher half-life (warmer and wetter climate where carbon degrades at a faster rate) and zero percent recycling of construction wood, whereas there are lower CO_2_ and CH_4_ emissions for disposal in Yukon (CO_2_: 0.10 kg C m^−3^, CH_4_: 0.13 kg C m^−3^) (long half-life with very slow degradation in cold dry climates) and Nova Scotia (high wood recycling rates). As shown in Fig. 4c and as was also shown in Fig. 3, the building lifetime also has a big effect in how the emissions are delayed, with fewer cumulative emissions taking place with higher building lifespans due to the prolonged storage of wood during the use phase.

**Fig. 6:**
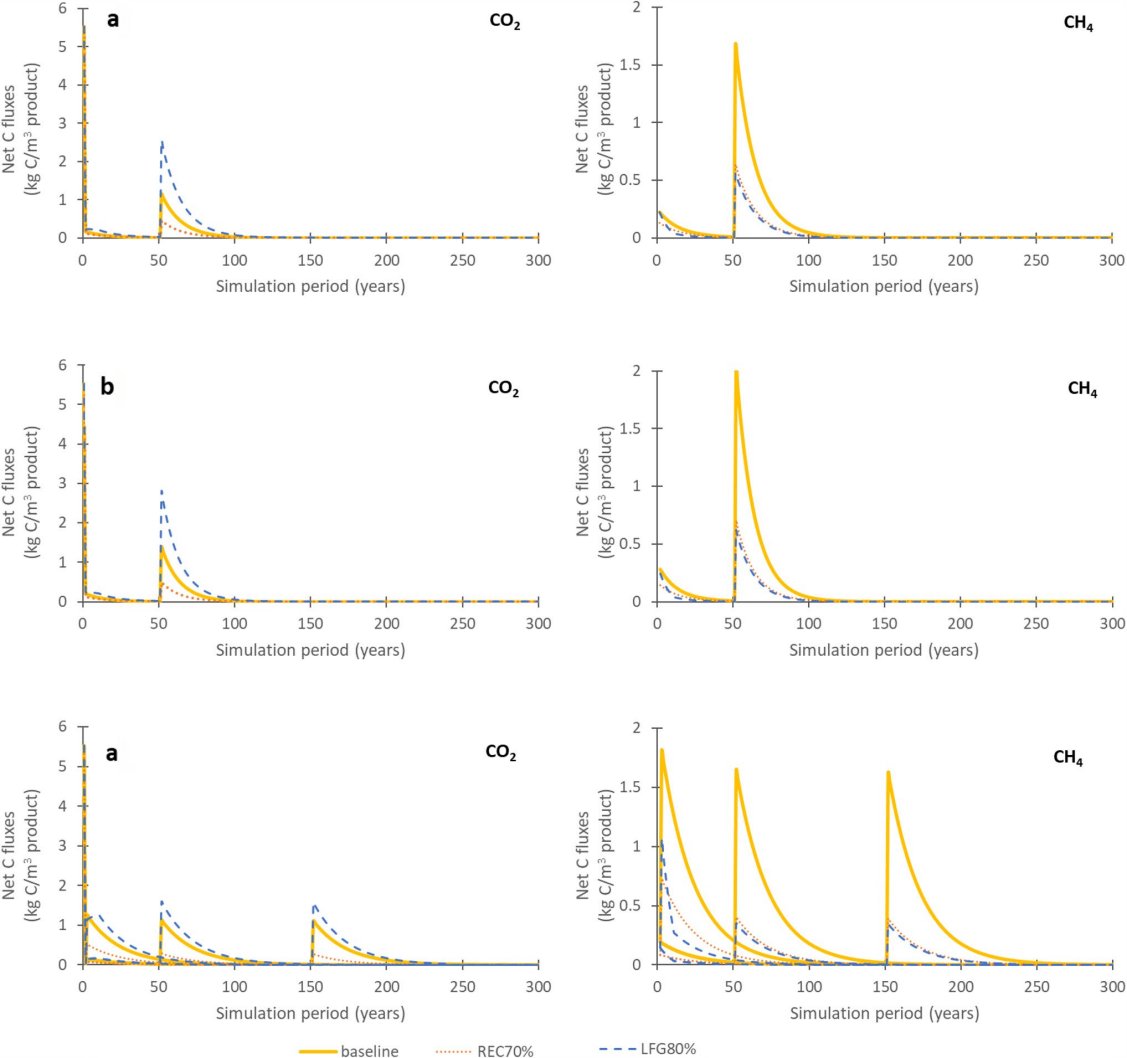
Net carbon fluxes (kg C/m^3^ product) of CO_2_ and CH_4_, comparing increased recycling policy (REC70% scenario) and increased landfill gas collection (LFG80% scenario) to the baseline scenario for lumber across **a** high recycling rates (Nova Scotia), **b** low landfill half-lives (British Columbia) and **c** different building lifespans (Ontario). BL1 = building lifespan 1 year, BL50 = building lifespan 50 years, BL150 building lifespan 150 years

The application of an increased construction and demolition waste recycling policy (REC70%) and an increased landfill gas collection policy (LFG80%) are compared in terms of CO_2_ and CH_4_ fluxes (as kg C) (Fig. 6). Results are shown in three cross-sections using lumber with building lifespan of 50 years as a default, such as to highlight the cases where differences between scenarios will most pronounced: a) high recycling rates (Nova Scotia), b) low landfill half-lives (British Columbia) and c) different building lifespans (Ontario). Since the policy scenarios change the recycling rates and landfill gas capture rates, the three cross-sections (a, b, c) were chosen in order to be able to best highlight the effects of the policy scenarios relative to the baseline scenario.

**Fig.7:**
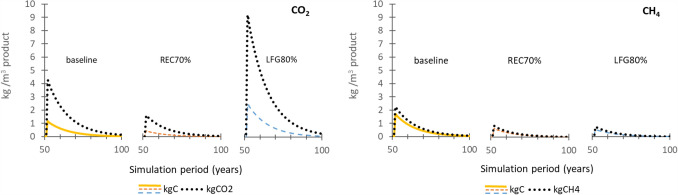
Excerpt of Fig. 6a (50–100 years) curves comparing kg C results with kg CO_2_ and kg CH_4_, for each baseline, REC70% scenario and LFG80% scenario curves

In general, increased construction and demolition waste recycling policy (REC70%) and an increased landfill gas collection policy (LFG80%) have a noticeable influence on the results (Fig. 6). The initial emissions released as CO_2_ from sawmilling and construction site wastes in year 1 are identical across all scenarios, which is due to the very recent implementations of the policy scenarios, which do not yet have an effect on net carbon fluxes. In terms of CO_2_ emissions, the increased landfill-gas capture scenario (LFG80%) has the largest C fluxes, as methane is captured, transformed into energy and ultimately flared to CO_2_ emissions. For CH_4_ emissions, the baseline scenario has the highest C fluxes and REC70% and LFG80% scenarios have quite similar and lower net C fluxes due to a either a decease in materials going to landfill (REC70%) or an increased capture of CH_4_ (LFG80%). While the results in Fig. 6 report the life cycle inventory in terms of kg C, an exerpt of Fig. 6a is also reported in Fig. 7 in terms of kg CO_2_ and kg CH_4_.

Due to the stoichiometric ratios between C and CO_2_ and CH_4_ (44/12 and 16/12, respectively), the emissions curves reported in terms of kg CO_2_ and kg CH_4_ are higher than the per kg C curves, particularly in the case of CO_2_. It is important to stress that these emissions are still at the inventory level and would require emissions characterisation (LCIA) to be expressed in terms of kg CO_2_-eq.

When recycling rates increase (REC70%, Fig. 6), lower net C fluxes than the baseline scenario results for both CO_2_ and CH_4_ emissions (end-of-life peaks of 0.3–0.5 kg C m^−3^ and cumulative emissions of 13.4–22.1 kg C m^−3^ for CO_2_—see Supplementary Material 2), due to less wood being sent to landfill. Instead, the recycling of this wood shifts the accountability of the carbon onto other product systems. Though it would seem that an increase in landfill gas capture (LFG80%, Fig. 5) only shifts CH_4_ fluxes to CO_2_ fluxes as methane is captured and is combusted, CH_4_ emissions have a much higher global warming potential than CO_2_ (25 kg CO_2_-eq kg^−1^ CH_4_ vs. 1 kg CO_2_-eq.·kg^−1^ CO_2_, Ciais et al. [70]). As such, increased landfill gas collection would reduce overall impacts on climate change at the life cycle impact level, namely from the conversion of CH_4_ to CO_2_ but also from potential fossil energy substitution that could take place from the generation of energy from landfill gas.

The choice of building life has a slight effect on the amplitude of the initial post-demolition emission peaks (Fig. 6c), which as discussed previously, differs between CO_2_ and CH_4_ emissions. For CO_2_, the baseline scenario has a peak of 1.3 kg C m^−3^ with building demolition after year 1, but only peaks at 1.1 kg C m^−3^ at demolition after 50 and 150 years. In the case of demolition at year 1, the CO_2_ peak is only recorded at year 3, a delay that can be accounted for by the time required for landfilled wood to begin decomposition. In contrast, LFG80%, has a peak of 1.1 kg C m^−3^ for a building lifespans of 1 year, but increases to 1.6 kg C m^−3^, for building lifespans of 50 and 150 years due to the increased CO_2_ from CH_4_ combustion. This shift is most evident in comparison with the CH_4_ results for LFG80%, where the initial post-demolition emission peak at demolition after 1 year is 1.1 kg C m^−3^, then drops off to 0.4 and 0.3 kg C m^−3^ after 50 and 150 years once the policy landfill gas capture targets have been reached. In terms of cumulative emissions, the baseline scenario has constant CO_2_ and CH_4_ emissions for all building lifespans (33.9 and 40.6 kg C m^−3^, respectively). Similar to the initial post-demolition emission peaks, the application of the policy scenarios changes the cumulative emissions totals from one building lifespan to another. For REC70%, for example, cumulative CO_2_ emissions are 17.4 kg C m^−3^ at BL1, vs. 13.6 kg C m^−3^ at BL50 and 13.4 kg C m^−3^ at BL150, while CH_4_ emissions are 16.4 kg C m^−3^ at BL1, vs. 10.8 kg C m^−3^ at BL50 and 10.6 kg C m^−3^ at BL150. The dynamic nature of recycling and landfill gas capture rates, means that the timing of the building demolition relative to the waste management practices in place at that given time, has impacts on the resulting emissions. For example, when the wood is sent to waste management treatment after 1 year of use, the cumulative net carbon fluxes of LFG80% are similar to those of the baseline. In the first year of LFG80% (2020), the methane capture is still the same as it is in the baseline scenario. LFG80% only begins to distinguish itself from the baseline as landfill gas capture rates begin to increase, as as they gradually approach 80% methane capture over a period of 10 years.

## Discussion

As would be expected, the results show that biogenic carbon emissions are delayed through the storage of carbon in buildings. Keeping the carbon stored in the technosphere by postponing the eventual disposal of wood products has a number of advantages. First, the climate may soon hit a tipping point, meaning that small changes in radiative forcing from emissions could trigger a strong response in the dynamics of the climate, irreparably changing its state [71]. As such, it would be beneficial to avoid the release of as many emissions as possible in the short-term [24, 71]. Second, postponing the eventual disposal of wood products may allow for the capacity for recycling to increase and for recycling markets to improve. For example, the Canadian government is currently developing the Clean Fuel Standard, which, when implemented, will aim to reduce greenhouse gas emissions from fuels by 30% by 2030. Some of the fuel pathways currently considered are to be produced from wood residues—this could be a potential treatment pathway for wood construction and demolition waste [72]. Finally, it would allow for landfill gas capture rates to improve, specifically if gas is utilised for energy instead of simply flaring CH_4_ to CO_2_. As older landfills close, it may become attractive to move towards CRD-specific (construction, renovation and demolition) landfills that do not emit as much landfill gas [73], due the landfilling of dry materials only. Kelleher Environmental, Guy Perry and Associates [46] found that there are already a number of municipalities with CRD-specific landfill sites.

As was aluded to the in the previous paragraph, the temporary storage of wood in buildings has the potential for climate benefits. This is especially true when considering the timing of emissions over the entire life cycle of a wood building product. To consider the effects of storage, the temporally-differentiated carbon emissions profiles developed in this work would need to be characterised to climate change potential (in kg CO_2_-eq) using a temporal LCIA method, which could be done in future work. The chosen time horizon, the period of consideration for the emissions and environmental impacts of a proudct, are an important considering in LCA, especially those covering considering long-life products and temporal emissions profiles. As such, the emissions or impacts of emissions occurring beyond the time horizon are not accounted for in the environmental impacts [74]. Typically in LCA, a time horizon of 100 years is used, although with the development of alternative global warming potential characterisation factors any time horizon can be chosen [75]. If, for example, a 100-year time horizon was chosen beginning in the year of the building construction and the wood remains in the building for 100 years, the end-of-life emissions of the wood beyond this point would not be included in the life cycle assessment. As such, as Levasseur [76] found, the choice of time horizon has an important effect on climate change results. In future research, the effects of selecting different time horizons could be explored with emission characterisation in LCIA.

In this paper, a cut-off approach was used for treating the multifunctionality of both the sawmill processes and the demolition waste. This approach may have some limitations, such as it does not considered additional benefits and burdens from valorizing co-products to be used in other product systems. That being said, the impacts from considering second product life cycles would be expected to vary significantly, based on the wide range of possible uses for products (bioenergy feedstock, mulch, fibreboard, reuse as a wood product, etc.). This could have significant contributions to the life cycle impacts of wood products. Further research is be warranted in order to validate a cut-off approach and to examine substitution effects of wood co-products.

The recycling of demolition wood generally decreases the carbon fluxes emitted by a given product, since the carbon is cut-off from the product life cycle and is shifted to a second product. However, its contributions depend on the particular circumstances of the building case. In the case of isolated northern regions, such Yukon and Northwest Territories, where low temperatures and precipitation result in very low landfill decomposition rates and subsequently low landfill emissions and where recycling markets are far away, landfilling may be preferable to recycling. However, this is dependent upon the system that is being modelled. While sending secondary wood to markets further south for recycling (e.g. in British Columbia) could mean more emissions in material transport than is avoided by sourcing virgin wood, local recycling could be overall beneficial, especially if it avoids having to to transport other materials to the north (e.g. housing materials, firewood). The evaluation of these types of effects could be included in future work on wood co-product substitution.

The uncertainties in this study are primarily associated with the proportions and fate of the sawmill co-products as well as the end-of-life fate of construction wood. The Athena reports referenced in this work are based on the mass balances and co-product fates of averaged surveyed sawmills, for which no statistical data is provided. As such, stastical ranges could not be calculated for each of the seven wood product types. Also due to the averaging of sawmill data and the lack of specific geographical content, any sawmill co-products sent to landfill were modelled using an average Canadian landfill. However, due to the small quantities of co-products treated via landfilling, having access to more information on the location of sawmills and sawmill landfills would likely not have much impact on the overall results. Very little literature exists on the fate of construction wood in Canada aside from the study referenced in this work [46], which is based on 2012 surveys of recycling and landfilling of wood and other construction materials. In addition, this study did not provide any statistical ranges or uncertainty analysis. Despite these shortcomings, this reference does provide a wide range of recycling rates across the country (0–50% depending on the province/territory), which allows for a wide range of testing for the upper and lower carbon flux limits of emissions profiles.

While this model has been developed in this research for the Canadian building context, the flexible nature of CBM-FHWP would allow modelling wood product life cycle cycles specific to other regions. In order to model for the context of another region, the following parameters would need to be adapted: the mass balance and fate of wood production at sawmill, the EOL fate of wood used in construction and background data on landfill half-lives. The adaptation of these elements would allow the model to track the fate of biogenic carbon for a region of the user’s choosing.

## Conclusion

In general, the results show that temporary storage of carbon in buildings delays emissions in the short-term. Most wood products have similar emissions profiles, although CLT and plywood deviate somewhat, primarily due to differences in product specifications. CLT has higher emissions from waste treatment at the sawmill (CO_2_: 0.16 kg C m^−3^ vs 0.02–0.06 kg C m^−3^, CH_4_: 0.24 kg C m^−3^ vs. 0.04–0.07 kg C m^−3^) due to a large proportion of off-specification product going to landfill. Plywood has lower demolition year emissions due to the main product containing a smaller proportion of carbon relative to wood inputs, as much of the wood mass goes to co-products in manufacturing.The province or territory where the building is constructed also has a large influence on the initial post-demolition emissions (CO_2_ range: 0.10–3.0 kg C m^−3^, CH_4_ range: 0.13–4.4 kg C m^−3^), due to variability in recycling rates and landfill gas decay rates. Higher recycling rates result in lower carbon fluxes, due to to fewer materials going to landfill causing CH_4_ and CO_2_ emissions. The coldest and driest regions have the longest landfill half-lives (347 years^−1^ in Yukon landfills), which result in a much slower degradation in the landfill and thus a shift of CH_4_ and CO_2_ emissions over time, closer to or beyond the time horizon relevant for the decision. The longer building lifepans have both the effect of delaying emissions and an effect on the amplitude of the initial C flux at the year of demolition.

The policy scenarios show the effects of implementing annual changes to the treatment of waste on the resulting carbon fluxes. Both the recycling policy (REC70%) and the landfill gas policy (LFG80%) showed significant changes in emissions after demolition, particularly for those building lifespans that extend beyond the policy target years for the scenarios. In the case of the landfill gas capture scenario, an increased capture shifts CH_4_ to CO_2_ as landfill gas is combusted through energy utilisation and flaring. The recycling policy reduces the overall CO_2_ and CH_4_ emissions as it shifts the carbon from the landfill to other product systems, that become accountable for the eventual carbon emissions.

This research demonstrates the use of a harvested wood product model that has been designed for the regional scale, to generate carbon fluxes for single wood products. The resulting carbon fluxes can be expressed as greenhouse emissions and be used to determine dynamic life cycle inventories for modelling the manufacturing, use and end-of-life phases of a cradle-to-grave LCAs of single wood products. More explicitly, the results could be integrated in an LCA tool, such as a building information model (BIM), where the biogenic carbon fluxes could be automatically modelled for a given wood product. Combining these temporally differentiated biogenic carbon fluxes for single wood products with the forestry emissions and removals inventory and dynamic life cycle impact assessment, would allow building designers to improve the relevance of climate change results and make informed choices on building material selection. The results could also be used to develop large scale scenarios of the building sector such to inform climate strategy.

## Supplementary information

ESM1 (272 kb)

ESM1 (22 kb)

ESM1 (13 kb)

ESM1 (2,290 kb)

ESM1 (2,281 kb)

ESM1 (2,277 kb)

ESM1 (2,284 kb)

ESM1 (2,262 kb)

ESM1 (2,272 kb)

ESM1 (2,258 kb)

## Data Availability

All key inputs and key outputs provided in the Supplementary Materials.

## Footnotes

1 1–10 years: annual increments, 10–50 years: 5-year increments, 50–150 years: 10-year increments.
